# Concurrent versus sequential use of trastuzumab and chemotherapy in early HER2+ breast cancer

**DOI:** 10.1007/s10549-020-05978-8

**Published:** 2020-10-28

**Authors:** Gwen M. H. E. Dackus, Katarzyna Jóźwiak, Elsken van der Wall, Paul J. van Diest, Michael Hauptmann, Sabine Siesling, Gabe S. Sonke, Sabine C. Linn

**Affiliations:** 1 Division of Molecular Pathology, Netherlands Cancer Institute, Plesmanlaan 121, 1066 CX Amsterdam, The Netherlands; 2 Department of Pathology, University Medical Center Utrecht, PO Box 85500, 3508 GA Utrecht, The Netherlands; 3 Department of Epidemiology and Biostatistics, Netherlands Cancer Institute, Plesmanlaan 121, 1066 CX Amsterdam, Netherlands; 4 Institute of Biostatistics and Registry Research, Brandenburg Medical School Theodor Fontane, Haus O, Fehrbelliner Straße 38, 16816 Neuruppin, Germany; 5 Division of Internal Medicine and Dermatology, University Medical Center Utrecht, Hpn Q05.4300, Heidelberglaan 100, 3584 CX Utrecht, The Netherlands; 6 Department of Research and Development, Netherlands Comprehensive Cancer Organisation The Netherlands, PO Box 19079, 3501 DB Utrecht, The Netherlands; 7 Department of Health Technology & Services Research, Technical Medical Center, University of Twente, PO Box 217, 7500 AE Enschede, The Netherlands; 8 Department of Medical Oncology, Netherlands Cancer Institute, Plesmanlaan 121, 1066 CX Amsterdam, The Netherlands

**Keywords:** Breast cancer, Human epidermal growth-factor receptor 2, Adjuvant treatment, Trastuzumab, Concurrent, Sequential

## Abstract

**Purpose:**

The addition of trastuzumab to adjuvant chemotherapy has improved the outcome of human epidermal growth-factor receptor 2 (HER2)-positive breast cancer. Uncertainty remains about the optimal timing of trastuzumab treatment. Therefore, we compared long-term outcome after concurrent versus sequential treatment, in a population-based setting, using data from the nationwide Netherlands Cancer Registry.

**Methods:**

We identified 1843 women diagnosed in The Netherlands from January 1st 2005 until January 1st 2008 with primary, HER2-positive, T_1-4_N_any_M_0_ breast cancer who received adjuvant chemotherapy and trastuzumab. Kaplan–Meier survival estimates and Cox regression were used to compare recurrence-free survival (RFS) and overall survival (OS) between women who received trastuzumab concurrently with versus sequentially after chemotherapy. Hazard ratios (HR) were adjusted for age, year of diagnosis, grade, pathological T-stage, number of positive lymph nodes, ER-status, PR-status, socio-economic status, radiotherapy, hormonal therapy, ovarian ablation, and type of chemotherapy.

**Results:**

After a median follow-up of 8.2 years, RFS events had occurred in 224 out of 1235 (18.1%) concurrently treated women and 129 out of 608 (21.2%) sequentially treated women (adjusted-HR 0.91; 95% confidence interval (CI) 0.67–1.24; *P* = 0.580). Deaths occurred in 182/1235 (14.7%) concurrently treated women and 104/608 (17.1%) sequentially treated women (adjusted-HR 0.92; 95% CI 0.65–1.29; *P* = 0.635).

**Conclusions:**

The results of this population-based study are consistent with earlier randomized trials, demonstrating a non-significant difference in outcome for concurrently treated women compared to those who were treated sequentially, suggesting both options are justified.

**Electronic supplementary material:**

The online version of this article (10.1007/s10549-020-05978-8) contains supplementary material, which is available to authorized users.

## Background

The introduction of trastuzumab, a monoclonal antibody targeting the extracellular domain of the human epidermal growth-factor receptor 2 (HER2), revolutionized the treatment of women with HER2+ breast cancer, who were among those with the poorest prognosis. Several studies, conducted in the adjuvant setting, showed impressive improvements in long-term outcome with the addition of trastuzumab to adjuvant chemotherapy [1–7]. Trastuzumab has, therefore, been incorporated in both national and international guidelines for the adjuvant treatment of HER2+ breast cancer [8, 9].

Most women receive trastuzumab in a concurrent treatment schedule. This is largely based on the results from the second NCCTG-N9831 phase-III trial interim-analysis, which showed a better disease free survival (DFS) with a concurrent rather than a sequential schedule (hazard ratio (HR), 0.77; 99.9% confidence interval (CI), 0.53 to 1.11), despite the fact that the results were not statistically significant at the pre-specified boundaries for interim-analysis [1].

NCCTG-N9831 was the only trial directly comparing adjuvant trastuzumab treatment sequences until the recent publication of the combined SIGNAL/PHARE trials [1, 10]. In SIGNAL/PHARE the likelihood of receiving either sequential or concurrent treatment depended on year of inclusion, with a split before and after 2011, the year in which the NCCTG-N9831 interim-analyses was published [10]. To account for this non-random treatment allocation a propensity score methodology was applied. The adjusted comparison showed no significant difference in overall survival (OS)(HR 1.01; 95% CI 0.86–1.19) and DFS (HR 1.08; 95% CI 0.96–1.21) between patients who were treated with a concurrent versus sequential regimen [10].

Both the NCCTG-N9831 interim-analyses and combined SIGNAL/PHARE trials found no significant difference between concurrent and sequential treatment regimens.

The aim of our study was, therefore, to re-evaluate whether there is a difference in outcome between patients who received trastuzumab sequentially after versus concurrently with chemotherapy using real world data from a large, population-based cohort derived from the Netherlands Cancer Registry (NCR), consisting of patients treated prior to the publication of the NCCTG-N9831 results.

## Methods

### Patient selection

We used the NCR to identify women who were diagnosed in The Netherlands, from January 1st 2005 until but not including January 1st 2008, with a primary HER2+, T_1-4_N_any_M_0_ breast cancer for which they received any form of both chemotherapy and trastuzumab treatment. Immunohistochemistry for estrogen receptor (ER), progesterone receptor (PR) and HER2 was performed at the local pathology laboratories as part of normal diagnostic workflow. This information was extracted from the pathology reports by NCR datamanagers. ER and PR were considered positive when ≥ 10% of tumor cells stained positive. Tumors were considered HER2 positive when scoring 3 + on immunohistochemistry or showing amplification on in situ hybridization or Multiplex ligation-dependent probe amplification [11–14].

Information on socio-economic status (SES—low, intermediate, high) was provided to us by the NCR who obtained this information from statistics Netherlands. Statistics Netherlands base their indicator on average income, percentage of people with low income, educational level and unemployment rates at the four digit postal code level of a womans residency at the time of diagnosis [15, 16].

Vital status was obtained through linkage with the municipal population registry. Information on cause of death and the development of subsequent second primary cancers was not available. NCR datamanagers returned to the patient files to retrieve information on disease recurrences as these are not routinely registered in the Dutch cancer registry.

### Statistical analysis

Patients were grouped by trastuzumab treatment sequence, concurrent or sequential, based on treatment start- and stopdates. We considered patients to be treated concurrently if they received two or more trastuzumab administration before the end of chemotherapy. All other patients were considered sequentially treated. Differences in baseline characteristics between sequentially and concurrently treated patients were assessed using chi-square, Fisher’s exact and linear-by-linear tests for categorical variables and *t*-tests for continuous variables.

The endpoints of our study were recurrence free survival (RFS) and OS. RFS time was calculated from date of diagnosis until death from any cause or invasive ipsilateral, local, regional or distant recurrence, whichever occurred first. OS time was calculated from date of diagnosis until death from any cause [17]. Patients lost to follow-up and those without a RFS or OS event were censored at the date of last follow-up.

The Kaplan–Meier method and Cox proportional hazards regression were used to assess RFS and OS. Univariable (unadjusted) and multivariable (adjusted) Cox regression models were used to estimate hazard ratios with 95% confidence intervals to compare treatment groups. We used age, year of diagnosis, grade, pathological T-stage, number of positive lymph nodes, ER-status, PR-status, SES, radiotherapy, hormonal therapy, ovarian ablation and type of chemotherapy received as covariates in our multivariable models. Proportional hazards assumptions were tested for the main, sequential-concurrent treatment effect, using Schoenfeld residuals. The assumptions were satisfied.

Sensitivity analysis were performed using three alternative definitions for concurrent and sequential treatment to check whether using different cut-offs significantly influenced our results. Besides OS and RFS we also calculated distant recurrence free interval (DRFI), defined as distant recurrence or death from breast cancer [17]. Because information on cause of death was lacking in our database we used death following a distant recurrence as a surrogate for death from breast cancer. In addition, due to regional differences in sequential and concurrent treatment, an alternative Cox model incorporating province of residence at time of diagnosis was constructed. Moreso, propensity score matching was performed to reduce possible bias using a nearest neighbor matching approach without replacement in a 1:1 ratio with a caliper of 0.05. Except for chemotherapy, all covariates from the main Cox model were included in a logistic regression model used to obtain propensity scores. Cox models for RFS and OS including matched pair treatment groups were then adjusted for propensity score and chemotherapy. Furthermore, we investigated whether trastuzumab treatment benefit differed by ER-status, nodal status and year of diagnosis, using likelihood ratio testing of interaction terms. Lastly, all analyses were repeated in node positive women and in women who were treated with anthracyclines and taxanes only.

All data were analyzed using R version 3.5.3 and packages ‘coin’, ‘lmtest’, ‘prodlim’, and ‘survival’ [18–21].

## Results

### Study population

**Fig. 1:**
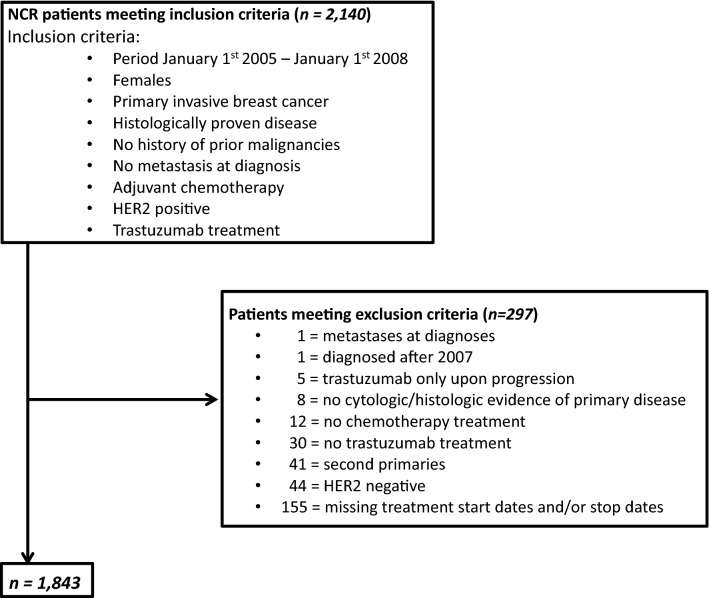
Flowchart of patient selection and inclusion from the Netherlands Cancer Registry (NCR) database. *HER2* human epidermal growth-factor receptor 2, *NCR* Netherlands Cancer Registry

The NCR identified 2140 potentially eligible women. We excluded 297/2140 (13.9%) women, mostly because of missing treatment start and/or stop dates, precluding classification of the treatment sequence. Hence, 1843 women were included in our study population (Fig. 1).

Baseline characteristics are shown for the entire study population and by trastuzumab treatment sequence (Table 1). In total, 67.0% (1235/1843) of the women in our cohort received trastuzumab concurrently with chemotherapy, while 33.0% (608/1843) received trastuzumab sequentially, following chemotherapy. The proportion of concurrently treated women increased over time from 53.8% (279/519) in 2005 to 76.1% (437/574) in 2007 (*P* < 0.001, linear-by-linear test) (Table 1, Online Resource 1). Median age at breast cancer diagnosis was 49 years (range 21–74 years) and socio-economic status was medium–high in 71.3% (1314/1843) of women. Most women had tumors that were T_2_ or smaller (86.5%; 1594/1843), node positive (62.8%; 1158/1843), grade 3 (62.3%; 1149/1843) and ER positive (53.3%; 983/1843) (Table 1). The majority of women received radiotherapy (71.1%; 1310/1843) and endocrine treatment (55.3%; 1020/1843) consisting of tamoxifen, an aromatase inhibitor or one of the two followed by the other. The chemotherapy schedule contained an anthracycline in 91.9% (1694/1843) of all women. In 72.3% (1332/1843) the anthracycline was combined with a taxane, either concurrent or sequential. This treatment approach was used more often in women who received trastuzumab concurrently compared to sequentially, 87.8% (1084/1235) versus 40.8% (248/608), respectively (*P* < 0.001) (Table 1).

**Table 1:** Baseline characteristics of 1843 Dutch patients with HER2+ breast cancer according to trastuzumab-chemotherapy treatment sequence

	Total cohort *N* = 1843	Sequential *N* = 608	Concurrent *N* = 1235	*P*
Median age in years (range)	49 (21–74)	49 (21–73)	49 (21–74)	*0.254*

	Total cohort *N* = 1843		Sequential *N* = 608		Concurrent *N* = 1235		*P*
	*N*	%	*N*	%	*N*	%	
Age (years)							
< 50	*942*	51.1	*305*	50.2	*637*	51.6	*0.602*
≥ 50	*901*	48.9	*303*	49.8	*598*	48.4	
Year of diagnosis							
2005	*519*	28.2	*240*	39.5	*279*	22.6	< *0.001*
2006	*750*	40.7	*231*	38.0	*519*	42.0	
2007	*574*	31.1	*137*	22.5	*437*	35.4	
Grade							
1	*40*	2.2	*15*	2.5	*25*	2.0	*0.139*
2	*430*	23.3	*154*	25.3	*276*	22.3	
3	*1149*	62.4	*355*	58.4	*794*	64.4	
Unknown	*224*	12.1	*84*	13.8	*140*	11.3	
Pathological T-stage							
pT1	*756*	41.0	*246*	40.5	*510*	41.3	*0.258*
pT2	*838*	45.5	*280*	46.0	*558*	45.2	
pT3	*83*	4.5	*36*	5.9	*47*	3.8	
pT4	*14*	0.8	*4*	0.7	*10*	0.8	
Unknown	*152*	8.2	*42*	6.9	*110*	8.9	
Positive lymph nodes							
0	*685*	37.2	*216*	35.5	*469*	38.0	*0.664*
1–3	*706*	38.3	*235*	38.7	*471*	38.1	
4–9	*299*	16.2	*102*	16.8	*197*	16.0	
> 10	*146*	7.9	*53*	8.7	*93*	7.5	
Unknown	*7*	0.4	*2*	0.3	*5*	0.4	
ER							
Negative	*838*	45.5	*263*	43.3	*575*	46.6	
Positive	*983*	53.3	*334*	54.9	*649*	52.5	
Unknown	*22*	1.2	*11*	1.8	*11*	0.9	*0.261*
PR							
Negative	*1050*	57.0	*339*	55.7	*711*	57.6	*0.218*
Positive	*730*	39.6	*257*	42.3	*473*	38.3	
Unknown	*63*	3.4	*12*	2.0	*51*	4.1	
SES							
Low	*517*	28.0	*172*	28.2	*345*	28.0	*0.749*
Medium	*740*	40.2	*237*	39.0	*503*	40.7	
High	*574*	31.1	*195*	32.1	*379*	30.7	
Unknown	*12*	0.7	*4*	0.7	*8*	0.6	
Radiotherapy							
No	*533*	28.9	*166*	27.9	*367*	29.7	*0.308*
Yes	*1310*	71.1	*442*	72.1	*868*	70.3	
Hormonal therapy							
No	*820*	44.5	*256*	42.1	*564*	45.7	*0.229*
Yes	*1020*	55.3	*351*	57.7	*669*	54.1	
AI	*665*		*229*		*436*		
AI/tamoxifen	*238*		*88*		*150*		
Tamoxifen	*117*		*34*		*83*		
Unknown	*3*	0.2	*1*	0.2	*2*	0.2	
Ovarian ablation							
No	*1462*	79.3	*481*	79.1	*981*	79.4	*0.302*
Yes	*381*	20.7	*127*	20.9	*254*	20.6	
LHRH agonist	*207*		*68*		*139*		
Surgery	*91*		*25*		*66*		
Both	*83*		*34*		*49*		
Chemotherapy^a^							
Anthracycline-based	*362*	19.6	*348*	57.1	*14*	1.1	< *0.001*
Anthracycline/taxane-based	*1332*	72.3	*248*	40.8	*1084*	87.9	
Taxane-based	*49*	2.7	*4*	0.7	*45*	3.6	
Carboplatin-based	*90*	4.9	*1*	0.2	*89*	7.2	
Unknown	*10*	0.5	*7*	1.2	*3*	0.2	

All *P* values < 0.05 were considered statistically significant

*A* doxorubicin, *AI* aromatase inhibitor, *C* cyclophosphamide, *Cb* carboplatin, *E* epirubicin, *ER* estrogen receptor, *F* 5-fluorouracil, *H* trastuzumab, *HER2*+ human epidermal growth-factor receptor 2 positive, *LHRH* luteinizing-hormone-releasing hormone, *P* paclitaxel, *PR* progesterone receptor, *SES* socio-economic status, *T* docetaxel

^a^Anthracycline-based schedules: (F)AC/(F)EC [7], anthracycline/taxane-based schedules: AC(dd)-P/AC(dd)-T/TAC/AT [2, 3, 7], taxane-based schedules: PH/TH [31], carboplatin-based schedules: TCbH/PCbH/(F)AC/(F)EC-PCH [3, 32, 33]

### Recurrence free survival

**Fig. 2:**
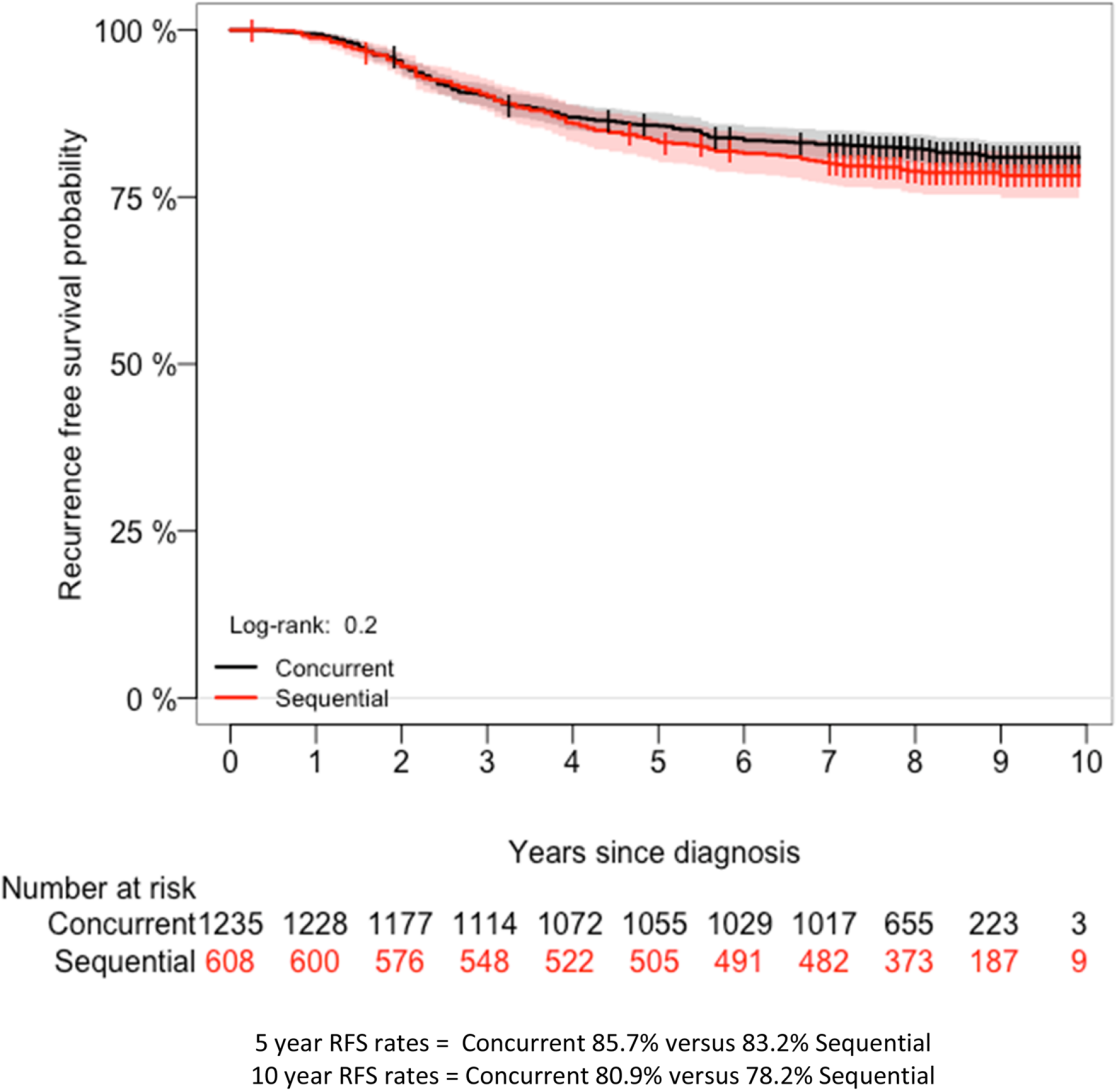
Kaplan–Meier curves showing RFS of 1843 HER2+ breast cancer patients according to trastuzumab-chemotherapy treatment sequence. *HER2* human epidermal growth-factor receptor 2, *RFS* recurrence-free survival

We observed 353 RFS events during a median follow-up of 8.1 years (range 0.3–9.9 years). Of these events, 19.9% (129/608) occurred in sequentially treated women and 18.1% (224/1235) in women who received trastuzumab concurrently with chemotherapy (Table 2). In both groups, distant metastases were the most frequently observed RFS event, followed by local recurrences and death (Online Resource 2). The observed difference in RFS between concurrently and sequentially treated women was not significant (5-year RFS 85.4% versus 83.1%; *P*_*log-rank*_ = 0.2—unadjusted-HR 0.85, 95% CI 0.68–1.06; *P* = 0.16) (Fig. 2, Table 2). When adjusted for age, year of diagnosis, grade, T-stage, number of positive lymph nodes, ER-status, PR-status, SES, radiotherapy, hormonal therapy, ovarian ablation and the type of chemotherapy received, the HR between concurrent and sequentially treated women remained unchanged (adjusted-HR 0.91, 95% CI 0.67–1.24; *P* = 0.580) (Table 2).

**Table 2:** Hazard ratios (HR) for recurrence-free survival in 1843 Dutch patients with HER2+ breast cancer

	Events	Unadjusted-HR (95% CI)	*P*	Adjusted-HR (95% CI)	*P*
Trastuzumab sequence^a^					
Sequential	129	1.00		1.00	
Concurrent	224	0.85 (0.68–1.06)	*0.16*	0.91 (0.67–1.24)	*0.580*
Age (years)					
< 50	173	1.00		1.00	
≥ 50	180	1.08 (0.88–1.33)	*0.435*	0.90 (0.71–1.14)	*0.403*
Year of diagnosis					
2005	103	1.00		1.00	
2006	136	0.93 (0.72–1.21)	*0.618*	0.86 (0.66–1.13)	*0.302*
2007	114	1.10 (0.84–1.45)	*0.460*	1.05 (0.79–1.40)	*0.692*
Grade					
Grade 1	4	0.50 (0.18–1.34)	*0.171*	0.62 (0.23–1.68)	*0.350*
Grade 2	75	0.90 (0.69–1.17)	*0.439*	0.91 (0.69–1.20)	*0.525*
Grade 3	216	1.00		1.00	
Unknown	58	1.46 (1.09–1.95)	*0.010*	1.64 (1.16–2.33)	*0.005*
Pathological T-stage					
T1	114	1.00	*0.009*	1.00	
T2	169	1.37 (1.08–1.73)	< *0.001*	1.17 (0.92–1.50)	*0.187*
T3	33	3.28 (2.22–4.83)	< *0.001*	1.96 (1.30–2.96)	*0.001*
T4	10	8.47 (4.43–16.18)	*0.367*	4.30 (2.13–8.68)	< *0.001*
Unknown	27	1.21 (0.79–1.84)		0.78 (0.48–1.25)	*0.311*
Positive lymph nodes					
0	87	1.00		1.00	
1–3	118	1.34 (1.01–1.77)	*0.037*	1.39 (1.04–1.85)	*0.022*
4–9	84	2.44 (1.80–3.29)	< *0.001*	2.48 (1.79–3.45)	< *0.001*
> 10	62	4.14 (2.99–5.74)	< *0.001*	3.86 (2.70–5.52)	*0.001*
Unknown	2	2.41 (0.59–9.79)	*0.218*	2.23 (0.53–9.37)	*0.293*
ER					
Positive	152	0.60 (0.48–0.74)	< *0.001*	0.86 (0.51–1.44)	*0.582*
Negative	197	1.00		1.00	
Unknown	4	0.72 (0.27–1.95)	*0.528*	0.80 (0.27–2.37)	*0.691*
PR					
Positive	100	0.55 (0.43–0.70)	< *0.001*	0.72 (0.53–0.99)	*0.045*
Negative	239	1.00	*0.852*	1.00	
Unknown	14	0.95 (0.55–1.62)		0.94 (0.52–1.69)	*0.837*
SES					
Low	98	1.07 (0.81–1.41)	*0.631*	1.13 (0.85–1.50)	*0.388*
Medium	150	1.15 (0.89–1.47)	*0.274*	1.11 (0.86–1.44)	*0.391*
High	103	1.00		1.00	
Radiotherapy					
Yes	261	1.00		1.00	
No	92	0.84 (0.66–1.07)	*0.170*	1.20 (0.92–1.58)	*0.171*
Hormonal therapy					
Yes	159	0.61 (0.49–0.75)	< *0.001*	0.86 (0.51–1.44)	*0.579*
No	193	1.00		1.00	
Ovarian ablation					
Yes	47	0.55 (0.40–0.75)	< *0.001*	0.76 (0.52–1.09)	*0.140*
No	306	1.00		1.00	
Chemotherapy					
Anthracyclines	77	1.21 (0.94–1.57)	*0.134*	1.19 (0.83–1.69)	*0.337*
Anthracyclines/taxane	235	1.00		1.00	
Taxanes	13	1.60 (0.91–2.80)	*0.096*	1.47 (0.82–2.62)	*0.187*
Other	26	1.82 (1.21–2.74)	*0.004*	1.67 (1.06–2.65)	*0.027*
Unknown	2	1.22 (0.30–4.93)	*0.774*	1.16 (0.28–4.81)	*0.830*

All *P* values < 0.05 were considered statistically significant

*CI* confidence interval, *ER* estrogen receptor, *HER2*+ human epidermal growth-factor receptor 2 positive, *HR* hazard ratio, *PR* progesterone receptor, *RFS* recurrence free survival, *SES* socio-economic status

^a^Patients were considered concurrently treated if they received more than one trastuzumab administration before the end of chemotherapy. All other patients were considered sequentially treated

### Overall survival

**Fig. 3:**
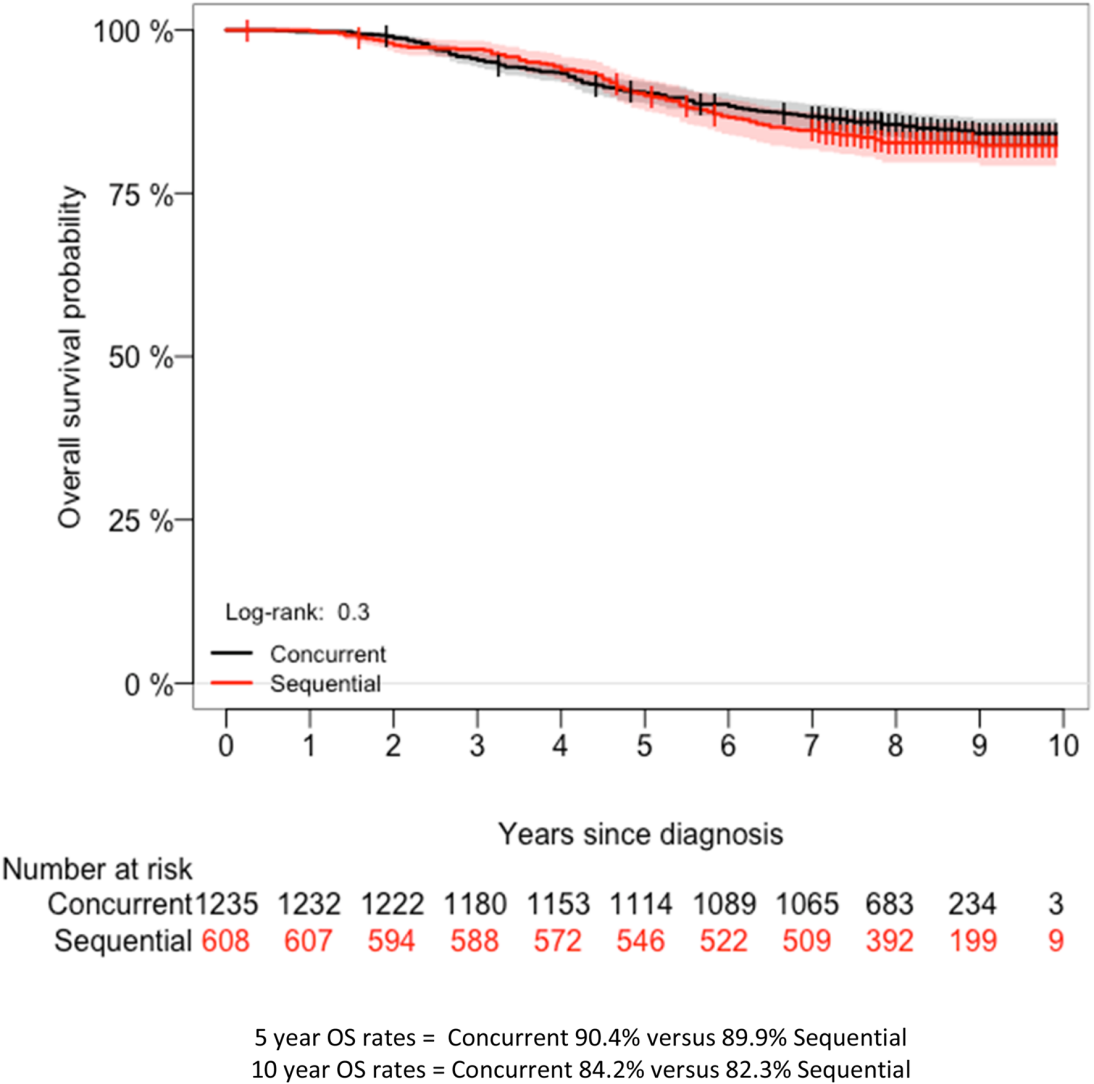
Kaplan–Meier curves showing OS of 1843 HER2+ breast cancer patients according to trastuzumab-chemotherapy treatment sequence. *HER2* human epidermal growth-factor receptor 2, *OS* overall survival

During a median follow-up of 8.2 years (range 0.2–10 years), 286 deaths occurred. Of these events 17.1% (104/608) of deaths occurred in sequentially treated women compared to 14.7% (182/1235) in women who received trastuzumab concurrently with chemotherapy. Again, we found no significant difference between women who received trastuzumab concurrently with chemotherapy when compared to sequentially following chemotherapy (5-year OS 90.2% versus 89.8%; *P*_*log-rank*_ = 0.3—unadjusted-HR 0.87, 95% CI 0.68–1.11; *P* = 0.269) (Fig. 3, Table 3). When corrected for the abovementioned characteristics the HR for OS between concurrently and sequentially treated women remained unchanged (adjusted-HR 0.92, 95% CI 0.65–1.29; *P* = 0.635) (Table 3).

**Table 3:** Hazard ratios (HR) for overall survival (OS) in 1843 Dutch patients with HER2+ breast cancer

	Events	Unadjusted-HR (95% CI)	*P*	Adjusted-HR (95% CI)	*P*
Trastuzumab sequence^a^					
Sequential	104	1.00		1.00	
Concurrent	182	0.87 (0.68–1.11)	*0.269*	0.92 (0.65–1.29)	*0.635*
Age (years)					
< 50	132	1.00		1.00	
≥ 50	154	1.24 (0.98–1.57)	*0.062*	1.08 (0.84–1.40)	*0.636*
Year of diagnosis					
2005	81	1.00		1.00	
2006	110	0.98 (0.73–1.31)	*0.913*	0.90 (0.67–1.22)	*0.641*
2007	95	1.22 (0.90–1.66)	*0.181*	1.12 (0.81–1.55)	*0.407*
Grade					
Grade 1	2	0.30 (0.07–1.22)	*0.094*	0.38 (0.09–1.54)	*0.177*
Grade 2	59	0.86 (0.64–1.16)	*0.348*	0.86 (0.63–1.17)	*0.355*
Grade 3	177	1.00		1.00	
Unknown	48	1.44 (1.05–1.99)	*0.023*	1.53 (1.05–2.25)	*0.027*
Pathological T-stage					
T1	91	1.00		1.00	
T2	130	1.31 (1.00–1.72)	*0.044*	1.09 (0.83–1.44)	*0.523*
T3	33	4.17 (2.79–6.21)	< *0.001*	2.40 (1.57–3.69)	< *0.001*
T4	9	8.97 (4.52–17.83)	< *0.001*	4.42 (2.11–9.28)	< *0.001*
Unknown	23	1.27 (0.80–2.01)	*0.300*	0.82 (0.48–1.38)	*0.460*
Positive lymph nodes					
0	67	1.00		1.00	
1–3	96	1.42 (1.04–1.94)	*0.027*	1.51 (1.09–2.08)	*0.012*
4–9	67	2.48 (1.77–3.49)	< *0.001*	2.55 (1.76–3.71)	< *0.001*
> 10	54	4.64 (3.24–6.64)	< *0.001*	4.35 (2.93–6.46)	< *0.001*
Unknown	2	3.37 (0.82–13.79)	*0.090*	3.44 (0.81–14.61)	*0.093*
ER					
Positive	113	0.52 (0.41–0.66)	< *0.001*	0.79 (0.45–1.38)	*0.418*
Negative	169	1.00		1.00	
Unknown	4	0.85 (0.31–2.30)	*0.757*	1.03 (0.34–3.12)	*0.951*
PR					
Positive	79	0.54 (0.41–0.70)	< *0.001*	0.87 (0.60–1.24)	*0.444*
Negative	196	1.00		1.00	
Unknown	11	0.90 (0.49–1.65)	*0.739*	0.93 (0.47–1.81)	*0.838*
SES					
Low	81	1.02 (0.76–1.39)	*0.858*	1.07 (0.78–1.45)	*0.667*
Medium	115	1.01 (0.76–1.33)	*0.924*	0.97 (0.73–1.29)	*0.873*
High	88	1.00		1.00	
Radiotherapy					
Yes	213	1.00		1.00	
No	73	0.83 (0.63–1.08)	*0.170*	1.22 (0.90–1.66)	*0.187*
Hormonal therapy					
Yes	119	0.53 (0.42–0.67)	< *0.001*	0.74 (0.42–1.30)	*0.304*
No	166	1.00		1.00	
Ovarian ablation					
Yes	34	0.48 (0.33–0.69)	< *0.001*	0.77 (0.50–1.18)	*0.233*
No	252	1.00		1.00	
Chemotherapy					
Anthracyclines	62	1.17 (0.88–1.56)	*0.265*	1.11 (0.75–1.65)	*0.588*
Anthracyclines/taxanes	191	1.00		1.00	
Taxanes	8	1.13 (0.55–2.30)	*0.726*	1.00 (0.48–2.07)	*0.994*
Other	24	2.01 (1.31–3.08)	*0.001*	1.87 (1.15–3.04)	*0.011*
Unknown	1	0.69 (0.09–4.96)	*0.717*	0.69 (0.09–5.02)	*0.716*

All *P* values < 0.05 were considered statistically significant

*CI* confidence interval, *ER* estrogen receptor, *HER2*+ human epidermal growth-factor receptor 2 positive, *HR* hazard ratio, *OS* overall survival, *PR* progesterone receptor, *SES* socio-economic status

^a^Patients were considered concurrently treated if they received more than one trastuzumab administration before the end of chemotherapy. All other patients were considered sequentially treated

### Sensitivity analysis

Similar RFS and OS results were obtained in sensitivity analyses using alternative definitions for sequential and concurrent treatment (Online Resource 3). Analyses for DRFI showed 267 events, 89/608 (14.6%) occurring in sequentially treated women compared to 178/1235 (14.4%) women who received trastuzumab concurrently with chemotherapy. The observed difference in DRFI between concurrently and sequentially treated women was not significant (five-year DRFI 87.8% versus 87.2%; *P*_*log-rank*_ = 0.9—djusted-HR 0.96, 95% CI 0.67–1.36; *P* = 0.833) (Online Resource 4).

We also observed no heterogeneity in the treatment effect by ER-status, nodal status and year of diagnosis for both RFS and OS (all *P*-values > 0.05). Moreover, adding province as a covariate to our Cox models did not significantly change results (Online Resource 5). Furthermore, when we repeated the analyses excluding 692 women with N_x_ or N_0_ disease, the HRs for OS at 5 years (adjusted-HR 0.80 95% CI 0.53–1.20) and RFS (adjusted-HR 0.83 95% CI 0.58–1.17) were comparable to the HR of the main analyses. Likewise, when analyses were repeated, only in women who were treated with anthracyclines and taxanes (*n* = 1332), HRs for OS (adjusted-HR 0.85 95% CI 0.59–1.21) and RFS (adjusted-HR 0.82 95% CI 0.59–1.13) were similar to the those obtained in the main analyses. Cox models adjusted for propensity scores yielded similar results confirming our main conclusions.

## Discussion

In our large population-based cohort of patients with early HER2-positive breast cancer, we found no significant difference in RFS and OS after concurrent versus sequential treatment with chemotherapy and trastuzumab; however, a consistent but non-significant numerical difference in favor of concurrent use was seen for all endpoints.

Most clinical trials that established the role of trastuzumab in the adjuvant setting, compared either the concurrent or sequential chemotherapy-trastuzumab regimen to a control arm containing no trastuzumab [1–7]. Studies comparing the timing of trastuzumab administration are sparse. The ALTTO study contained sequential and concurrent treatment arms both alone and in combination with lapatinib, but did not directly compare the two treatment sequences [22]. The NCCTG-N9831 trial and combined SIGNAL/PHARE trials are therefore the only trials that compared sequential to concurrent trastuzumab in the adjuvant setting. In the SIGNAL/PHARE trial 5572 women received trastuzumab according to physician’s choice [10], Similar to our study, 65.5% of women in the SIGNAL/PHARE trial were treated concurrently and 34.5% sequentially. After a median follow-up of 58 months, no difference in OS (HR 1.01; 95% CI 0.86–1.19) and DFS (HR 1.08; 95% CI 0.96–1.21) was observed when comparing sequential to concurrent treatment [10]. Results from the NCCTG-N9831 interim-analysis on the other hand are more in line with our results, with a slight improvement in DFS when comparing trastuzumab concurrently with versus sequentially after chemotherapy (HR 0.77; 99.9% CI 0.53–1.11) [1]. The observed difference in DFS was not significant as it did not cross the prespecified O’Brien-Fleming boundary of significance [1]. The definitive joint analysis of the NCCTG-N9831 and NSABP-B31 left out the sequential treatment arm (arm B) from the NCCTG-N9831 trial and compared doxorubicin/cyclophosphamide followed by trastuzumab given concurrently with paclitaxel to a control arm without trastuzumab [23].

Differences between the SIGNAL/PHARE and NCCTG-N9831 studies may be caused by the non-random treatment allocation in the SIGNAL/PHARE study after publication of the NCCTG-N9831 interim-analyses results [10]. However, this was accounted for using a propensity score methodology. Our cohort also observed a significant increase in the proportion of concurrently treated women over time, from 53.8% (279/519) in 2005 to 76.1% (437/574) in 2007 (*P* < 0.001, linear-by-linear test). Although our cohort originates from before full publication of the NCCTG-N9831 data, its initiation preceeds both the presentation and publication of the first interim-analyses results in 2005 [1, 2, 10].

When looking at the number of women included in our cohort, it seems that there is an imbalance in HER2+ women diagnosed and hence included, with 519 and 750 included women in 2005 and 2006, respectively, compared to 574 women in 2007. However, In the early years of HER2 testing assay quality and interpretation varied considerably between laboratories, leading up to false positive test results in 18% of patients included in large, randomized trials like the NSABP-B31 trial [24]. The variation in number of women included is therefore, most likely a reflection of this high false positive rate rather then a true imbalance in patient inclusion. It wasn’t until 2007 that the American Society for Clinical Oncolgy published a guideline for the recommendation of HER2 testing in breast cancer [25].

Many studies, including SIGNAL/PHARE and the NCCTG-N9831 trial, used DFS as one of their study endpoints. As information on the occurence of second primary cancers was lacking for the women in our cohort we had to use RFS instead [17]. DFS time would have been shorter for women who experienced a second primary cancer in the absence of, or prior to a locoregional or distant recurrence. With a median age of 49 years at diagnosis, however, women in our cohort are relatively young and the incidence of secondary primary cancers low. We therefore think that the results for RFS are comparable to those for DFS.

The concurrent treatment groups in both SIGNAL/PHARE and our study may be enriched with high-risk patients since women received trastuzumab treatment according to physicians choice. However, we observed no variation in baseline characteristics between women who received trstuzumab concurrently with versus sequentially after chemotherapy (Table 1).

Most trials were originally enriched for node positive (N +), high-risk, patients. In our study, 37.2% (685/1843) of the patients were N0. Therefore, we investigated whether trastuzumab sequence benefit differed by nodal status, to ensure that the N0 patients did not influence the observed overall treatment effect. We found no heterogeneity in the treatment effect by nodal status (data not shown).

Anthracyclines are especially effective in HER2-positive breast cancer [26–28]. Sequential schedules are preferred as anthracyclines administered concurrently with trastuzumab cause high rates of symptomatic heart failure [28] In the SIGNAL/PHARE trial 33.3% of sequentially treated women received an anthracycline without a taxane compared to 0.8% in the concurrent treatment group [10]. Similarly, in the sequential treatment group of our study 57.1% (348/608) of women received an anthracycline compared to 1.1% (14/1235) in the concurrent treatment group. Since the addition of taxanes to anthracycline-based adjuvant treatment schedules improved the outcome of breast cancer patients in general, regimens for HER2-positive breast cancer patients were developed where trastuzumab was started sequentially after the anthracycline-based part of the regimen and concurrently with a taxane [2, 29, 30]. An alternative strategy was to give six instead of 3–4 anthracycline-based chemotherapy cycles followed sequentially by trastuzumab, which had a low rate of overt heart failure [5]. This may explain why taxanes are given in concurrent treatment groups. In our cohort 1332 women received chemotherapy containing both anthracyclines and taxanes, 1084/1235 (87.8%) concurrently treated women compared to 248/608 (40.8%) sequentially treated women. We repeated the analyses in women who were treated with anthracyclines and taxanes only and found HRs for OS (adjusted-HR 0.85 95% CI 0.53–1.20) and RFS (adjusted-HR 0.83 95% CI 0.58–1.17) that were similar to those obtained in the main analyses meaning that women who received both anthracyclines and taxanes do not derive a differential treatment benefit from trastuzumab treatment sequence.

As cause of death was not known for the women in our cohort we used death following a distant recurrence as a surrogate for death from breast cancer. A substantial number of women in our cohort, therefore, may have died from causes other than breast cancer. In the sequential group 17.8% (23/129) of RFS events consisted of death in the absence of breast cancer recurrence, compared to 12.1% (27/224) in concurrently treated women (Online Resource 2). Although these numbers may seem large, they only pertain to 3.8% (23/608) of the sequentially and 2.2% (27/1235) of the concurrently treated women, respectively. Since neither the clinical SIGNAL/PHARE and NCCTG-N9831 trials nor our population-based study showed superiority of the concurrent over sequential treatment schedule, additional factors like comorbidities and side effects gain importance when choosing a patient’s treatment schedule. The slight imbalance in deaths in the absence of breast cancer recurrence, observed between the sequentially and concurrently treated women in our study, may therefore reflect the clinicians’ preference for a sequential treatment scheme in patients suffering from comorbidities. Unfortunately, we do not have access to reliable information on comorbidities or performance status in our data set to correct for this. We did calculate DRFI to see whether this imbalance impacted outcome and found results similar to the main analyses (Online Resource 3).

In addition, our analysis may have suffered from immortal time bias since only women who did not experience early events, before trastuzumab initiation, were included in our cohort. However, we do not believe that this has impacted our results since there is no reason to believe that the duration of immortal time or the occurrence of early events varies between women who received trastuzumab concurrently with versus sequentially after chemotherapy. In addition, the time between diagnosis and treatment initiation is often relatively short and the incidence of early events low.

Lastly, the results presented in this paper are based on data derived from a population-based cohort. As a result, women were not randomized and received treatment according to the guidelines at time of diagnosis. Although we performed multivariable adjustment for potential confounders, confounding may still play a role in our observational study. Therefore, propensity score matching was performed in an attempt to further reduce any possible confounding effects. The observed change in HR for concurrent versus sequentially treated women, of less than 10% points, was small and therefore confirmed our main conclusions.

## Conclusions

In conclusion, although we observed a slight improvement in both OS and RFS in women who received concurrent trastuzumab compared to those treated sequentially, results did not reach statistical significance. Therefore, both treatment approaches are justified and decisions may be made on an individual patient basis where the shorter duration of the concurrent regimen must be balanced with potential treatment-related toxicities and pre-existing comorbidities. A future meta analysis, using all published studies to date, may be useful in providing a more precise estimate of the true difference in outcome between concurrently and sequentially treated women with HER2+ breast cancer.

## Electronic supplementary material

Below is the link to the electronic supplementary material.

Supplementary file2 (DOCX 49 kb)

Supplementary file3 (DOCX 31 kb)

Supplementary file4 (DOCX 107 kb)

Supplementary file5 (DOCX 91 kb)

Supplementary file6 (DOCX 113 kb)

Supplementary file1 (DOCX 45 kb)

## Data Availability

The data that support the findings of this study are available from the Netherlands Cancer Registry, hosted by the Netherlands Comprehensive Cancer Centre (IKNL) but restrictions apply to the availability of these data, which were used under license for the current study, and so are not publicly available. Data are, however, available from the authors upon reasonable request and with permission of The Netherlands Comprehensive Cancer Centre (IKNL).

## Abbreviations

A: Doxorubicin
AI: Aromatase inhibitor
C: Cyclophosphamide
Cb: Carboplatin
CI: Confidence interval
DFS: Disease free survival
DRFI: Distant recurrence free interval
E: Epirubicin
ER: Estrogen receptor
F: 5-Fluorouracil
H: Trastuzumab
HER2: Human Epidermal growth-factor Receptor 2
HR: Hazard ratio
LHRH: Luteinizing-hormone-releasing hormone
NCR: Netherlands Cancer Registry
N+: Node positive
OS: Overall survival
P: Paclitaxel
PR: Progesterone receptor
RFS: Recurrence free survival
SES: Socio-economic status
T: Docetaxel background
