# Supplementary material for: Concurrent versus sequential use of trastuzumab and chemotherapy in early HER2+ breast cancer
Source: Breast Cancer Res Treat. 2020 Oct 28;185(3):817–30. doi: 10.1007/s10549-020-05978-8 (PMC7921067; doi:10.1007/s10549-020-05978-8)
Supplement: Supplementary file 1 — Supplementary file2 (DOCX 49 kb) [file 10549_2020_5978_MOESM1_ESM.docx]

**Journal:** Breast Cancer Research and Treatment

**Concurrent versus sequential use of trastuzumab and chemotherapy in early HER2+ breast cancer**

Gwen MHE Dackus (g.dackus@nki.nl) ^a,b^, Katarzyna Jóźwiak (katarzyna.jozwiak@mhb-fontane.de) ^c,d^, Elsken van der Wall (E.vanderWall@umcutrecht.nl) ^e^, Paul J van Diest (P.J.vanDiest@umcutrecht.nl) ^b^, Michael Hauptmann (Michael.Hauptmann@mhb-fontane.de) ^c,d^, Sabine Siesling (S.Siesling@iknl.nl) ^f,g^, Gabe S Sonke* (g.sonke@nki.nl) ^h^, Sabine C Linn* (s.linn@nki.nl) ^a,b,h^

*These authors contributed equally

**Corresponding author:**

Prof. Sabine C Linn

Netherlands Cancer Institute, Department of Medical Oncology

Plesmanlaan 121, 1066CX Amsterdam, the Netherlands

Phone: +31-20-512 2951

Fax: +31-20-512 2572

E-mail: [s.linn@nki.nl](mailto:s.linn@nki.nl)

**ONLINE RESOURCE 1:** Trastuzumab-chemotherapy treatment sequence (concurrent or sequential) by year of diagnosis for 1,843 Dutch patients with Human Epidermal growth-factor Receptor 2 (HER2+) breast cancer


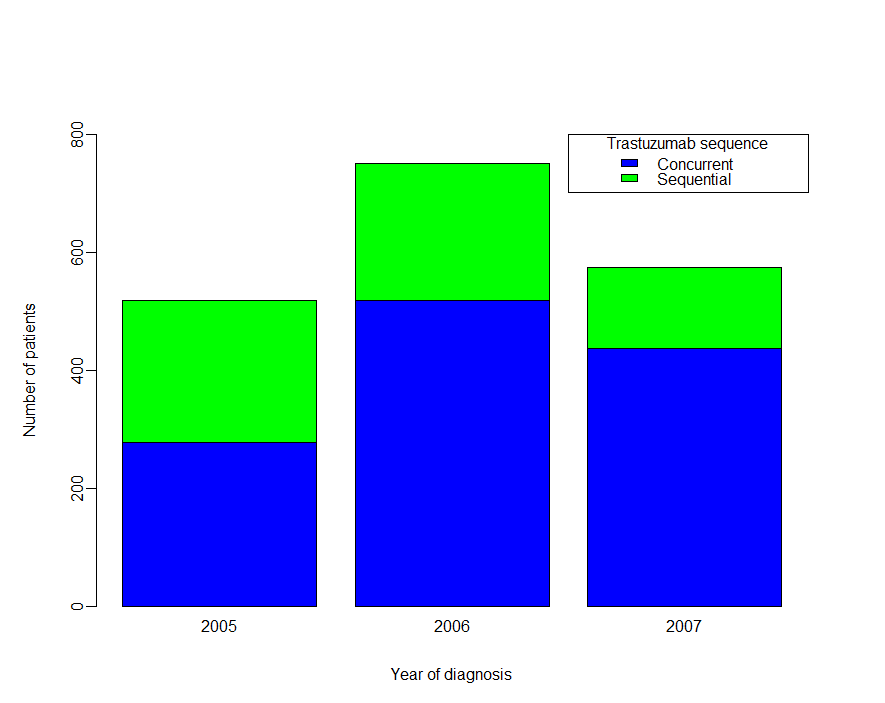


|  | **2005** | | **2006** | | **2007** | |
| --- | --- | --- | --- | --- | --- | --- |
|  | *n* | % | *n* | % | *n* | % |
| **Sequential** | *240* | 46.2 | *231* | 30.8 | *137* | 23.9 |
| **Concurrent** | *279* | 53.8 | *519* | 69.2 | *437* | 76.1 |
| ***Total*** | *519* | *100* | *750* | *100* | *574* | *100* |
