# Supplementary material for: Concurrent versus sequential use of trastuzumab and chemotherapy in early HER2+ breast cancer
Source: Breast Cancer Res Treat. 2020 Oct 28;185(3):817–30. doi: 10.1007/s10549-020-05978-8 (PMC7921067; doi:10.1007/s10549-020-05978-8)
Supplement: Supplementary file 2 — Supplementary file3 (DOCX 31 kb) [file 10549_2020_5978_MOESM2_ESM.docx]

**Journal:** Breast Cancer Research and Treatment

**Concurrent versus sequential use of trastuzumab and chemotherapy in early HER2+ breast cancer**

Gwen MHE Dackus (g.dackus@nki.nl) ^a,b^, Katarzyna Jóźwiak (katarzyna.jozwiak@mhb-fontane.de) ^c,d^, Elsken van der Wall (E.vanderWall@umcutrecht.nl) ^e^, Paul J van Diest (P.J.vanDiest@umcutrecht.nl) ^b^, Michael Hauptmann (Michael.Hauptmann@mhb-fontane.de) ^c,d^, Sabine Siesling (S.Siesling@iknl.nl) ^f,g^, Gabe S Sonke* (g.sonke@nki.nl) ^h^, Sabine C Linn* (s.linn@nki.nl) ^a,b,h^

*These authors contributed equally

**Corresponding author:**

Prof. Sabine C Linn

Netherlands Cancer Institute, Department of Medical Oncology

Plesmanlaan 121, 1066CX Amsterdam, the Netherlands

Phone: +31-20-512 2951

Fax: +31-20-512 2572

E-mail: [s.linn@nki.nl](mailto:s.linn@nki.nl)

**ONLINE RESOURCE 2**: Number and type of first recurrence free survival (RFS) event for all 1,843 Dutch patients with Human Epidermal growth-factor Receptor 2 positive (HER2+) breast cancer according to trastuzumab-chemotherapy treatment sequence^a^

|  | Sequential | | | Concurrent | | | |  |
| --- | --- | --- | --- | --- | --- | --- | --- | --- |
|  | ***N*** | **%** | **% sequential cohort** | | ***N*** | **%** | **% concurrent cohort** | |
| Local recurrence | *20* | 15.5 | 3.3 | | *33* | 14.7 | 2.7 | |
| Regional recurrence | *6* | 4.7 | 1.0 | | *13* | 5.8 | 1.1 | |
| Locoregional recurrence | *2* | 1.6 | 0.3 | | *1* | 0.4 | 0.1 | |
| Distant metastases | *78* | 60.4 | 12.8 | | *150* | 67.0 | 12.1 | |
| Death | *23* | 17.8 | 3.8 | | *27* | 12.1 | 2.2 | |
| Total number of events | *129* | 100 | 21.5 | | *224* | 100 | 18.1 | |

^a^ Number of RFS events that occurred first in the patient group and treatment arm as indicated. For example, in the women who received trastuzumab concurrent with chemotherapy, 224 RFS events occurred. In total, 150/224 (67.0%) RFS events involved distant metastases as first site, affecting 12.1% of all concurrently treated women in our cohort
