# Supplementary material for: Concurrent versus sequential use of trastuzumab and chemotherapy in early HER2+ breast cancer
Source: Breast Cancer Res Treat. 2020 Oct 28;185(3):817–30. doi: 10.1007/s10549-020-05978-8 (PMC7921067; doi:10.1007/s10549-020-05978-8)
Supplement: Supplementary file 3 — Supplementary file4 (DOCX 107 kb) [file 10549_2020_5978_MOESM3_ESM.docx]

**Journal:** Breast Cancer Research and Treatment

**Concurrent versus sequential use of trastuzumab and chemotherapy in early HER2+ breast cancer**

Gwen MHE Dackus (g.dackus@nki.nl) ^a,b^, Katarzyna Jóźwiak (katarzyna.jozwiak@mhb-fontane.de) ^c,d^, Elsken van der Wall (E.vanderWall@umcutrecht.nl) ^e^, Paul J van Diest (P.J.vanDiest@umcutrecht.nl) ^b^, Michael Hauptmann (Michael.Hauptmann@mhb-fontane.de) ^c,d^, Sabine Siesling (S.Siesling@iknl.nl) ^f,g^, Gabe S Sonke* (g.sonke@nki.nl) ^h^, Sabine C Linn* (s.linn@nki.nl) ^a,b,h^

*These authors contributed equally

**Corresponding author:**

Prof. Sabine C Linn

Netherlands Cancer Institute, Department of Medical Oncology

Plesmanlaan 121, 1066CX Amsterdam, the Netherlands

Phone: +31-20-512 2951

Fax: +31-20-512 2572

E-mail: [s.linn@nki.nl](mailto:s.linn@nki.nl)

**ONLINE RESOURCE 3**: Visual representation and adjusted hazard ratios (HRs) for recurrence free survival (RFS) and overall survival (OS) using alternative definitions for the trastuzumab-chemotherapy treatment sequence. The upper panel shows the main model.

(A) RFS and OS for sequential treatment (no overlap) and concurrent treatment (≥1 trastuzumab administrations before the end of chemotherapy).

(B) RFS and OS for sequential treatment (no overlap) and concurrent treatment (≥ 2 trastuzumab administrations before the end of chemotherapy), excluding 21 patients with one trastuzumab administration before the end of chemotherapy.

(C) RFS and OS for sequential treatment (no overlap) and concurrent treatment (≥ 3 trastuzumab administrations before the end of chemotherapy), excluding 109 patients with two or less trastuzumab administration before the end of chemotherapy.

**A**

**B**

**C**

**Sequential (3 weeks)**

**Concurrent (3 weeks)**

**Excluded**

**Chemotherapy**

A

|  | Number of patients | Events | HR^a^ RFS | 95% CI | *P* |
| --- | --- | --- | --- | --- | --- |
| **Trastuzumab sequence**  Sequential  Concurrent | 587  1,256 | 125  228 | 1.00  0.90 | 0.66-1.23 | *0.531* |

|  | Number of patients | Events | HR^a^ OS | 95% CI | *P* |
| --- | --- | --- | --- | --- | --- |
| **Trastuzumab sequence**  Sequential  Concurrent | 587  1,256 | 101  185 | 1.00  0.90 | 0.63-1.27 | *0.556* |

B

|  | Number of patients | Events | HR^a^ RFS | 95% CI | *P* |
| --- | --- | --- | --- | --- | --- |
| **Trastuzumab sequence**  Sequential  Concurrent | 587  1,235 | 125  224 | 1.00  0.91 | 0.67-1.25 | *0.590* |

|  | Number of patients | Events | HR^a^ OS | 95% CI | *P* |
| --- | --- | --- | --- | --- | --- |
| **Trastuzumab sequence**  Sequential  Concurrent | 587  1,235 | 101  182 | 1.00  0.91 | 0.64-1.29 | *0.617* |

C

|  | Number of patients | Events | HR^a^ RFS | 95% CI | *P* |
| --- | --- | --- | --- | --- | --- |
| **Trastuzumab sequence**  Sequential  Concurrent | 587  1,147 | 125  206 | 1.00  0.91 | 0.66-1.26 | *0.585* |

|  | Number of patients | Events | HR^a^ OS | 95% CI | *P* |
| --- | --- | --- | --- | --- | --- |
| **Trastuzumab sequence**  Sequential  Concurrent | 587  1,147 | 101  168 | 1.00  0.91 | 0.64-1.31 | *0.640* |

CI = confidence interval, HR = hazard ratio, OS = Overall Survival, RFS = Recurrence Free Survival

^a^ All Hazard Ratios were corrected for age, incidence year, grade, pathological T-stage, number of positive lymph nodes, ER-status, PR-status, SES, radiotherapy, hormonal therapy and ovarian ablation in accordance with our main analyses (tables 2 and 3).
