# Supplementary material for: Concurrent versus sequential use of trastuzumab and chemotherapy in early HER2+ breast cancer
Source: Breast Cancer Res Treat. 2020 Oct 28;185(3):817–30. doi: 10.1007/s10549-020-05978-8 (PMC7921067; doi:10.1007/s10549-020-05978-8)
Supplement: Supplementary file 4 — Supplementary file5 (DOCX 91 kb) [file 10549_2020_5978_MOESM4_ESM.docx]

**Journal:** Breast Cancer Research and Treatment

**Concurrent versus sequential use of trastuzumab and chemotherapy in early HER2+ breast cancer**

Gwen MHE Dackus (g.dackus@nki.nl) ^a,b^, Katarzyna Jóźwiak (katarzyna.jozwiak@mhb-fontane.de) ^c,d^, Elsken van der Wall (E.vanderWall@umcutrecht.nl) ^e^, Paul J van Diest (P.J.vanDiest@umcutrecht.nl) ^b^, Michael Hauptmann (Michael.Hauptmann@mhb-fontane.de) ^c,d^, Sabine Siesling (S.Siesling@iknl.nl) ^f,g^, Gabe S Sonke* (g.sonke@nki.nl) ^h^, Sabine C Linn* (s.linn@nki.nl) ^a,b,h^

*These authors contributed equally

**Corresponding author:**

Prof. Sabine C Linn

Netherlands Cancer Institute, Department of Medical Oncology

Plesmanlaan 121, 1066CX Amsterdam, the Netherlands

Phone: +31-20-512 2951

Fax: +31-20-512 2572

E-mail: [s.linn@nki.nl](mailto:s.linn@nki.nl)

**ONLINE RESOURCE 4:** Kaplan-Meier curves showing the distant recurrence free interval (DRFI) of 1,843 Dutch patients with Human Epidermal growth-factor Receptor 2 positive (HER2+) breast cancer according to trastuzumab-chemotherapy treatment sequence


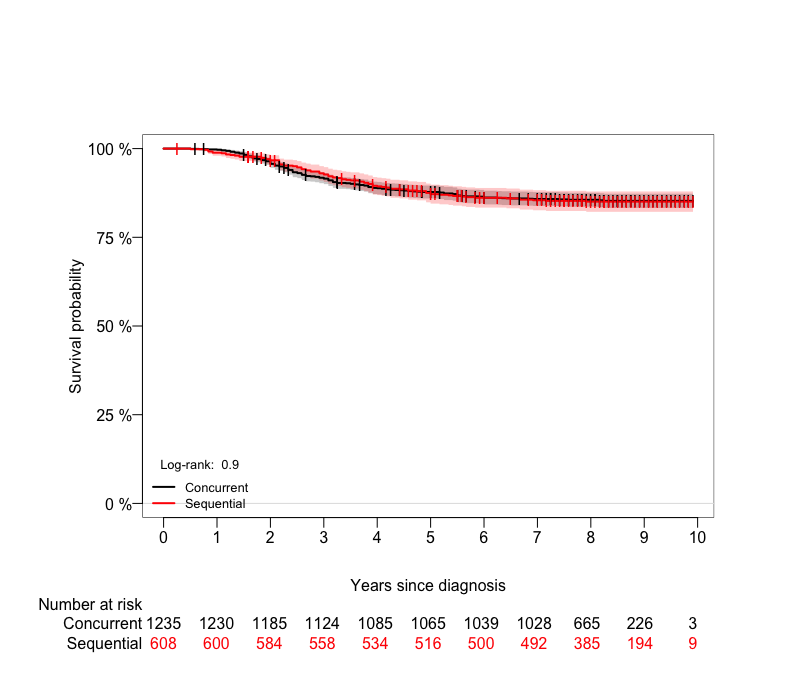


Adjusted-HR 0.92 (95% CI 0.65-1.31; *P=0.647*)

5 year DRFI rates = Concurrent 87.8% versus 87.2% Sequential

10 year DRFI rates = Concurrent 85.3% versus 85.0% Sequential

CI = Confidence interval, DRFI = Distant Recurrence Free Interval, HER2+ = Human Epidermal growth-factor Receptor 2 positive, HR = Hazard Ratio
