# Supplementary material for: Concurrent versus sequential use of trastuzumab and chemotherapy in early HER2+ breast cancer
Source: Breast Cancer Res Treat. 2020 Oct 28;185(3):817–30. doi: 10.1007/s10549-020-05978-8 (PMC7921067; doi:10.1007/s10549-020-05978-8)
Supplement: Supplementary file 5 — Supplementary file6 (DOCX 113 kb) [file 10549_2020_5978_MOESM5_ESM.docx]

**Journal:** Breast Cancer Research and Treatment

**Concurrent versus sequential use of trastuzumab and chemotherapy in early HER2+ breast cancer**

Gwen MHE Dackus (g.dackus@nki.nl) ^a,b^, Katarzyna Jóźwiak (katarzyna.jozwiak@mhb-fontane.de) ^c,d^, Elsken van der Wall (E.vanderWall@umcutrecht.nl) ^e^, Paul J van Diest (P.J.vanDiest@umcutrecht.nl) ^b^, Michael Hauptmann (Michael.Hauptmann@mhb-fontane.de) ^c,d^, Sabine Siesling (S.Siesling@iknl.nl) ^f,g^, Gabe S Sonke* (g.sonke@nki.nl) ^h^, Sabine C Linn* (s.linn@nki.nl) ^a,b,h^

*These authors contributed equally

**Corresponding author:**

Prof. Sabine C Linn

Netherlands Cancer Institute, Department of Medical Oncology

Plesmanlaan 121, 1066CX Amsterdam, the Netherlands

Phone: +31-20-512 2951

Fax: +31-20-512 2572

E-mail: [s.linn@nki.nl](mailto:s.linn@nki.nl)


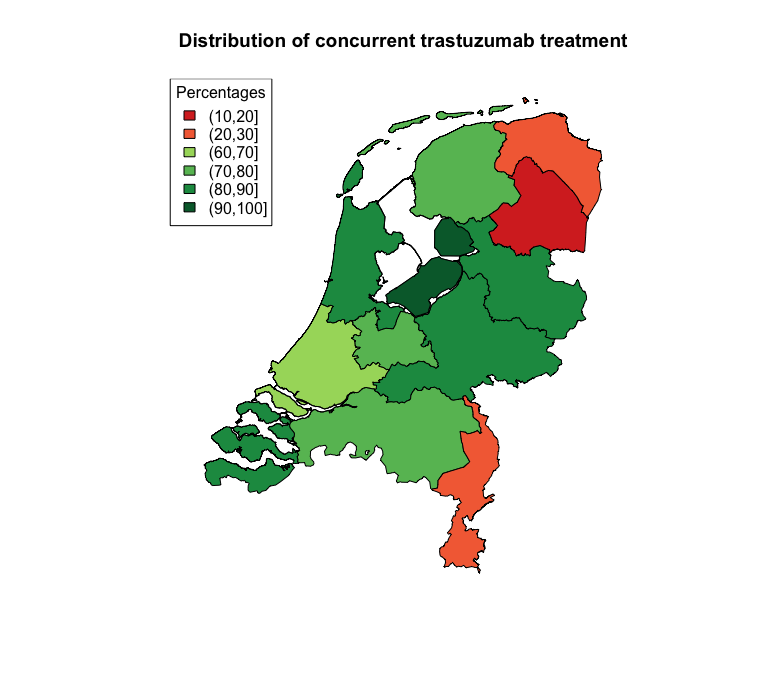


**ONLINE RESOURCE 5**: Two part figure showing the 1.843 Dutch women diagnosed with HER2+ breast cancer between 2005 and 2007 according to trastuzumab treatment sequence (concurrent or sequential) per province (lower panel) and the proportion of those women who received concurrent treatment (upper panel). The HRs for concurrently treated women versus sequentially treated women are shown for both overall survival (OS) and recurrence free survival (RFS) and were adjusted for the standard covariates supplemented by province

OS: adjusted-HR* 0.92 (95% CI 0.63-1.33)

RFS: adjusted-HR* 0.96 (95% CI 0.69-1.33)

| **Province** | **Sequential** | **%** | **Concurrent** | **%** | **Total** |
| --- | --- | --- | --- | --- | --- |
| Drenthe | 53 | 86.9 | 8 | 13.1 | 61 |
| Flevoland | 4 | 7.5 | 49 | 92.5 | 53 |
| Friesland | 21 | 23.1 | 70 | 76.9 | 91 |
| Gelderland | 30 | 15.1 | 169 | 84.9 | 199 |
| Groningen | 64 | 73.6 | 23 | 26.4 | 87 |
| Limburg | 125 | 79.6 | 32 | 20.4 | 157 |
| Noord-Brabant | 67 | 29.1 | 163 | 70.9 | 230 |
| Noord-Holland | 63 | 18.3 | 282 | 81.7 | 345 |
| Overijssel | 21 | 17.1 | 102 | 82.9 | 123 |
| Utrecht | 22 | 23.7 | 71 | 76.3 | 93 |
| Zeeland | 6 | 13.3 | 39 | 86.7 | 45 |
| Zuid-Holland | 107 | 30.8 | 240 | 69.2 | 347 |
| Unknown | 4 | 33.3 | 8 | 66.7 | 12 |

* The HRs were adjusted for age, year of diagnosis, grade, pathological T-stage, number of positive lymph nodes, ER-status, PR-status, SES, province, radiotherapy, hormonal therapy, ovarian ablation and type of chemotherapy
